# Characterization of blood-derived exosomal hTERT mRNA as a biomarker for colon cancer and Lynch syndrome

**DOI:** 10.3389/fonc.2022.962473

**Published:** 2022-09-20

**Authors:** Ido Laish, Zohar Levi, Hussein Mahajna, Ahmad Albshesh, Nir Horesh, Efraim Katz, Dan Feldman, Nadav Shinar, Orit Picard, Miri Yavzori, Ella Fudim, Pia Raanani, Tamar Berger, Hadar Goldvaser, Einat Beery, Orit Uziel

**Affiliations:** ^1^ Institute of Gastroenterology, Chaim Sheba Medical Center, Tel-Hashomer, Israel; ^2^ Sackler School of Medicine, Tel Aviv University, Tel Aviv, Israel; ^3^ Institute of Gastroenterology, Rabin Medical Center, Petah Tikva, Israel; ^4^ Department of Surgery and Transplantations B/C, Chaim Sheba Medical Center, Tel-Hashomer, Israel; ^5^ Institute of Gastroenterology, Meir Medical Center, Kfar-Saba, Israel; ^6^ Institute of Hematology, Davidoff Cancer Center, Rabin Medical Center, Petah Tikva, Israel; ^7^ The Felsenstein Medical Research Center, Rabin Medical Center, Petah Tikva, Israel; ^8^ Institute of Oncology, Shaare – Zedek Medical Center, Faculty of Medicine, Hebrew University, Jerusalem, Israel

**Keywords:** telomerases, exosomes, colon cancer, Lynch syndrome, genetic syndromes

## Abstract

**Background:**

Human telomerase reverse transcriptase (hTERT)- mRNA was shown to be elevated in exosomes derived from the sera of a variety of hematological and solid cancer patients. We aimed to evaluate its role as a diagnostic marker in patients with newly diagnosed colon cancer and in hereditary syndromes with predisposition to colon cancer.

**Methods:**

hTERT -mRNA levels were determined in serum-derived exosomes from 88 patients with colon cancer, 71 Lynch-syndrome carriers with unknown active malignancies and 50 healthy controls. Data, including demographics, background diseases, clinical data regarding tumor characteristics and genetic data, were retrieved data from medical files.

**Results:**

Patients with colon cancer had both higher exosomal hTERT mRNA levels and a higher proportion of patients with positive exosomal hTERT mRNA than controls (29.5% vs. 4%, respectively, P values < 0.001). Within the cancer group, patients with a metastatic disease had higher levels of telomerase mRNA than non-metastatic disease patients, and these levels correlated with CEA levels. Likewise, Lynch syndrome carriers had a higher proportion of positive exosomal hTERT mRNA than controls (21.1% vs. 4%, respectively, P value 0.008) but only a trend towards higher exosomal hTERT mRNA levels. Higher telomerase mRNA levels were not correlated with the mutated gene.

**Conclusions:**

Exosomal serum hTERT –mRNA levels are associated with metastatic colon cancer and were also demonstrated in a subset of Lynch syndrome carriers. Its significance as a biomarker for developing malignancy should be elucidated.

## Introduction

Colorectal carcinoma (CRC) is the second most common cause of cancer related death and the third most commonly detected cancer worldwide1 (1) . Currently, the only broadly used biomarker for CRC, chorio- embroginic antigen (CEA) has limited sensitivity and specificity and therefore there is an unmet need for novel biomarkers. Circulating mRNAs that encode human telomerase reverse transcriptase (hTERT) may be of particular interest in this setting. hTERT, the catalytic protein of the telomerase complex that synthetizes *de novo* telomere sequences, is considered a hallmark of cancer and is activated in more than 90% of cancer cells (2, 3). Its activity is essential for the endless proliferation and the perpetuation of the malignant clone and is correlated with hTERT mRNA (4). Several studies have demonstrated that hTERT mRNA level in tumors is an independent prognostic marker in various types of cancers (5, 6). Its serum levels are also increased in patients with various malignancies, including gastric (7), prostate (8), hepatocellular cancer (9) and also CRC (10), supporting the concept that circulating cell-free hTERT mRNA may be a promising non-invasive liquid biopsy related biomarker in clinical practice. Indeed, recent studies have shown its predictive and prognostic potential in CRC and in patients with rectal cancer undergoing chemo-radiotherapy (6, 11).

However, identifying of serum hTERT transcript is far from being accurate due to the unstable character of the transcripts. A more reliable, exosomal-based method, was recently reported by our group (12). Exosomes are small (30-150nm) membrane vesicles that originate from the endosomal membrane compartment. They contain mRNA, miRNA, DNA, proteins and lipids and are secreted by many cell types, including cancer cells, into the microenvironment and peripheral blood, thus fulfilling important roles in intercellular communications and perpetuation of carcinogenesis (13, 14). Cell-free mRNA is stabilized in exosomes and is considerably protected from digestion by extracellular RNases (15). We recently reported that hTERT mRNA is present in exosomes derived from all examined cancer cell lines (12), and it was also elevated in exosomes derived from the sera of many hematological and solid cancer patients with a variety of malignancies (16). Therefore, exosomal hTERT mRNA may potentially serve as a reliable ‘pan-cancer’ marker for the early detection of cancer.

The small number of patients with CRC in the cohort, however, precluded us from testing specific associations between exosomal hTERT mRNA levels and CRC and to correlate it with disease staging. In addition, exosomal hTERT mRNA’s levels in pre-malignant conditions, such as colonic polyps and hereditary CRC syndromes, were not tested. Lynch syndrome (LS) is an autosomal-dominant disorder caused by germline mutations in the DNA mismatch repair (MMR) genes (17). Although characterized by early-onset CRC and increased risk for other malignancies, there is considerable variation in malignancy susceptibility (18), probably related to genetic modifiers as well as environmental factors. It was previously shown that cancer-free MMR-deficient and MMR-proficient non-polyposis hereditary cases show distinct patterns of blood telomere lengths (19), suggesting the importance of the MMR system’s status on telomere length in hereditary cases, and that a genetic variant in the telomerase gene can modify the cancer risk in Lynch carriers (20).

Due to its potential diagnostic and pathogenic importance, we aimed to evaluate the use of peripheral blood exosomal hTERT mRNA as a valid diagnostic marker in patients with newly diagnosed CRC and LS carriers compared to healthy controls, and to assess its correlation with patient- and tumor-related factors.

## Methods

### Design and patient population

This was a case-control study conducted at the Sheba Medical Center (SMC) and Rabin Medical Center (RMC), both tertiary academic medical centers in Israel. We included patients with newly diagnosed biopsy-proven CRC, prospectively recruited between 6/2020 and 5/2021 in SMC (N=80), and CRC patients recruited between 5/2014 and 1/2015 in RMC (N=18), for which data was previously partially published (16). LS carriers, routinely followed by high risk clinics in these 2 medical centers, were recruited following a colonoscopy showing either no findings or benign polyps. Control patients were healthy individuals without a known hereditary cancer syndrome, who underwent colonoscopy for different etiologies (including screening, diagnostic, polyp surveillance or due to family history of cancer) showing either no findings or benign polyps. Both LS-carriers and controls were prospectively recruited in 2020-2021 at the same timeline of CRC patients. Baseline serum carcinoembryonic antigen (CEA) and exosomal hTERT mRNA were obtained from CRC patients before any surgical or oncological treatment.

Subsequent retrieved data from medical files included demographics, smoking habits, presence of diabetes, family history of CRC and aspirin use in all patients, and genetic information in LS carriers. For CRC patients, colonoscopy-, pathological- and cross-sectional imaging reports were explored for tumor localization, degree of differentiation and TMN staging, respectively. Immunohistochemistry for mismatch-repair (MMR) proteins was not available in all patients since routine screening for this was not yet included in Israel’s public health’s list of approved services (health basket). Immunohistochemistry for MMR was not performed on resected polyps in LS or control patients. Patients were excluded from the study if they were unable to provide informed consent or suffered from systemic active infectious diseases, autoimmune disorders (including inflammatory bowel disease) or other current known extra-colonic malignancies. Additionally, in order to avoid individuals with potential inherited polyposis syndrome, patients with multiple (≥ 10) adenomas were excluded. All patients signed an informed consent and the study was approved by the institutional ethics review boards.

### Exosomes purification

Exosomes were isolated from patients’ sera by using the Total Exosome Isolation Kit (Invitrogen, Carlsbad, CA, USA) according to the manufacturer’s instructions. Exosomes concentration and sizes were analyzed by using the NanoSight tracking device as previously described (12) and by electron microscopy (EM). Usually, 3-1010 exosomes were used for each hTERT mRNA analysis. Exosomal markers CD81 and CD19 were detected by flow cytometry.

### RNA purification and cDNA formation

RNA from exosomes was purified with the Total Exosome RNA and Protein Isolation Kit (Invitrogen) according to the provided manual. mRNA was reverse transcribed using the High-Capacity cDNA Reverse Transcription Kit (Applied Biosystems, Foster City, CA, USA) according to the manufacturer’s instructions.

### hTERT expression by real-time PCR

The expression of hTERT was measured relatively to that of the HPRT-1 as a reference gene. Gene amplification was executed using the following sets of primers (purchased from HyLabs, Rehovot, Israel). hTERT: forward, 5’- GTACTTTGTCAAGGTGGA-TGTGA-3’ and reverse, 5’-GCTGGAGGTCTGTCAAGGTAGAG-3’; HPRT-1: forward, 5’ TCAGGCAGTATAATCCAAAGATGGT-3’ and reverse, 5’-CTTCGTGGGGTCCTTTTCAC-3’. Polymerase chain reactions were prepared with the Taqman fluorophore-labelled primers (Applied Biosystems), run and analyzed on the Step One Detection System (Applied Biosystems). Reactions were performed for 50 cycles; a normal value (no expression of hTERT) was arbitrarily defined as 1 for further calculation purposes.

Exosomal hTERT mRNA was considered positive when Relative quantification (RQ) was > 1.2.

### Statistical analysis

Categorical variables were described as frequency and percentage and compared using the Chi-square test or Fisher’s exact test. Continuous variables were described as median and interquartile range (IQR) and comparisons between categories were done using Kruskal-Wallis and Mann–Whitney. Associations between telomerase- mRNA levels and continuous variables were assessed using Spearman’s correlation coefficient. All statistical tests were two sided and *p* < 0.05 was considered statistically significant. All statistical analyses were performed using SPSS (IBM SPSS Statistics for Windows, version 25, IBM Corp, Armonk, NY, USA).

## Results

Eighty eight patients with CRC [median age (range)- 68 (30–85)], 71 LS-carriers [median age (range)- 46 (27-77)], and 50 healthy controls [median age (range)- 68.5 (47-87)], were included in our cohort. There were 22 patients (44%) with adenomatous polyps in the control group and 17 LS-carriers (24%) with adenoma in their colonoscopy. Demographics and clinical characteristics are detailed in **Table 1**. LS- carriers were significantly younger and had lower rates of diabetes and aspirin use than the CRC patients and healthy controls, while having higher rate of family CRC history. There were no significant differences between CRC patients and healthy controls in these parameters.

**Table 1:** Baseline patient-, tumor- and genetic characteristics of the study groups.

	CRC group (N = 88)	Lynch syndrome carriers group (N= 71)	Control group (N= 50)	P value *	P value**	P value***
Age, median [IQR]	68.0 [57.2-73.7]	46 [38 – 61]	68.5 [60.7-70.0]	0.242	< 0.001	< 0.001
Male (%)	45 (51.1)	35 (49.3)	26 (52.0)	0.952	0.854	0.874
Smoking (%)	18 (20.5)	12 (16.9)	14 (28.0)	0.313	0.179	0.684
Diabetes (%)	17 (19.3)	5 (7.0)	12 (24.0)	0.516	0.015	0.036
Aspirin (%)	22 (25.0)	5 (7.0)	16 (32.0)	0.397	< 0.001	0.022
Family history of CRC (%)	9 (10.2)	51 (71.8)	3 (6.0)	0.130	< 0.001	<0.001
CRC staging (%)						
- I	21 (23.8)					
- II	25 (28.4)					
- III	17 (19.4)					
- IV	25 (28.4)					
Location (%)						
- Proximal	30 (34.0)					
- Distal	34 (38.6)					
- Rectal	24 (27.2)					
Differentiation (%)						
- Well/moderate	83 (94.3)					
- Poor	5 (5.7)					
MMR status						
- MMR-P	34 (38.6)					
- MMR-D	6 (6.8)					
- Unknown	48 (54.6)					
CEA > 5 ng/ml (%)	28 (31.8)					
MMR genes Mutation (%)						
- MLH1		9 (12.7)				
- MSH2		37 (52.1)				
- MSH6		20 (28.1)				
- PMS2		5 (0.1)				

CRC – Colorectal cancer; MMR-P – Mismatch repair proficient; MMR-D – Mismatch repair deficient.

* Between CRC and control groups.

** Between LS carriers and control groups.

***Between CRC and LS carriers groups.

Among the CRC patients, 25 (28.4%) had metastatic disease (stage IV) and most tumors (94.3%) had well/moderate differentiation. The MMR status was known for 40 tumors, of which 34 (85%) were MMR-proficient and 6 (15%) were MMR-deficient with MLH1/PMS2 loss of staining and positive BRAF mutation, which associates with a sporadic tumor. Among LS-carriers, the most common pathogenic variants were in the MSH2 (52.1%) and MSH6 (28.1%) genes, reflecting the high frequency of Ashkenazi-Jewish founder mutations in these genes (21).

### Association with exosomal hTERT mRNA levels

**Figure 1:**
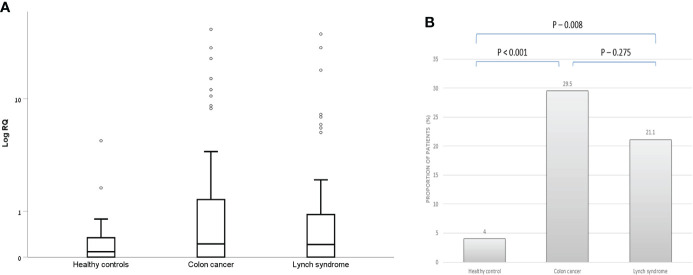
Exosomal hTERT mRNA levels in the study groups. **(A)** Box and Whisker plot of log hTERT mRNA median values and **(B)** percent of patients with positive hTERT mRNA values [Relative quantification (RQ) > 1.2].

There were 26 patients in the CRC group, 15 patients in the LS-carriers group and two patients in the control group, who had positive (RQ > 1.2) exosomal hTERT mRNA levels. As depicted in **Figure 1**, patients with CRC had both higher exosomal hTERT mRNA levels than controls [median (IQR) 0.22 (0.0- 1.48) vs. 0.08 (0.0- 0.34), respectively; P value – 0.01] and a higher proportion of patients with positive exosomal hTERT mRNA (29.5% vs. 4%, respectively, P values < 0.001). Likewise, LS carriers had higher proportions of positive exosomal hTERT mRNA than controls (21.1% vs. 4%, respectively, P value 0.008) but only a trend towards higher exosomal hTERT mRNA levels, [median (IQR) 0.21 (0.0- 0.96) vs. 0.08 (0.0- 0.34), respectively; P value – 0.11]. There were no statistically significant differences between CRC patients and LS carriers in both these measures (**Figures 1A, B**). None of the other demographic or clinical parameters in our cohort were associated with exosomal hTERT mRNA levels (**Table 2**).

**Table 2:** ssociation between patient- and tumor-characteristics and high exosomal hTERT mRNA in a univariate analysis.

Characteristic	Patients with high hTERT mRNA (%)	P value
Age		0.292
Sex - Male - Female	22 (20.8) 21 (20.4)	0.948
Smoking - Yes - No	8 (18.2) 35 (21.2)	0.659
Diabetes - Yes - No	8 (23.5) 35 (20.0)	0.641
Aspirin use - Yes - No	9 (23.1) 34 (20.0)	0.668
Family history of CRC - Yes - No	13 (20.6) 30 (20.5)	0.989
Tumor localization - Proximal - Distal - Rectal	5 (16.7) 12 (35.3) 9 (39.1)	0.141
Tumor differentiation - Well/moderate - Poor	24 (28.9) 2 (40)	0.580
MMR status - MMR-P - MMR-D	5 (14.7) 1 (16.6)	0.656
CEA level - ≤ 5 ng/ml - > 5 ng/ml	13 (21.7) 13 (48.1)	0.013

CRC, Colorectal cancer; MMR-P, Mismatch repair proficient; MMR-D, Mismatch repair deficient.

**Figure 2:**
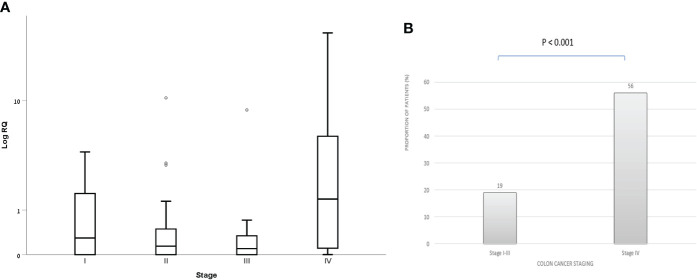
Exosomal hTERT mRNA levels in CRC patients according to disease staging **(A)** Box and Whisker plot of log hTERT mRNA median values and **(B)** percent of patients with positive hTERT mRNA values [Relative quantification (RQ) > 1.2].

Within the CRC group, patients with stage 4 had both higher exosomal hTERT mRNA levels than those in stage 1,2 and 3 [median (IQR) 1.38 (0.92- 7.41) vs. 0.29 (0.0- 1.64), 0.14 (0.0- 0.54) and 0.10 (0.0- 0.37), respectively; P value – 0.016] and a higher proportion of patients with positive exosomal hTERT mRNA (56.0% vs. 19.0% in stage 4 vs. 1-3, respectively, P values < 0.001) (**Figures 2A, B**). Higher CEA levels were also positively associated with higher exosomal hTERT mRNA levels [median (IQR) 0.71 (0.10- 3.03) vs. 0.15 (0.0- 0.78) in high and low CEA patients, respectively; P value – 0.005] and also with the proportion of patients with positive exosomal hTERT mRNA (48.1% vs. 21.7% in high and low CEA patients, respectively; P value – 0.013), although displaying a weak correlation (r- 0.328, P value - 0.02). Neither patient’s characteristics, including family history of cancer, nor tumor characteristics - including tumor localization, grade of differentiation and MMR status, were associated with exosomal hTERT mRNA levels (**Table 2**).

**Figure 3:**
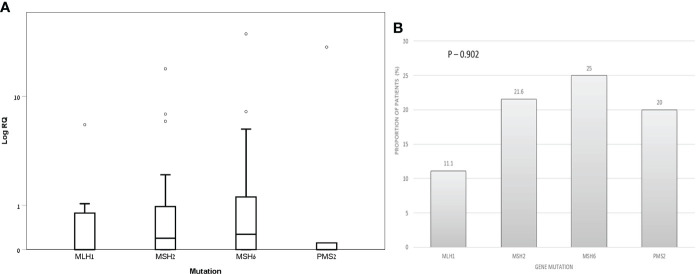
Exosomal hTERT mRNA levels in Lynch syndrome carriers according to genetic mutation **(A)** Box and Whisker plot of log hTERT mRNA median values and **(B)** percent of patients with positive hTERT mRNA values [Relative quantification (RQ) > 1.2].

Within the LS-carriers group, the median (IQR) exosomal hTERT mRNA levels were 0.0 (0.0- 0.91), 0.21 (0.0- 0.98), 0.27 (0.0- 1.56) and 0.0 (0.0- 11.44) in MLH1, MSH2, MSH6 and PMS2 mutation carriers, respectively, without significant difference (P value – 0.77) (**Figure 3A**). The proportion of patients with positive exosomal hTERT mRNA were also comparable between the groups (**Figure 3B**). Among LS carriers with positive exosomal hTERT mRNA, 9/15 (60%) individuals had previous CRC and 3/15 (20%) had other Lynch-associated cancer. Carriers with (N=17) and without (N=54) adenoma had both the same exosomal hTERT mRNA levels [median (IQR) 0.20 (0.0-0.86) vs. 0.21 (0.0- 0.98), respectively; P value – 0.92] and proportion of positive patients for hTERT mRNA (17.6% vs. 22.2%, respectively; P value – 0.75).

## Discussion

The detection of cancer-related RNA molecules in plasma, specifically cell-free circulating TERT mRNA, can be potentially used as a disease marker. In this study we showed that CRC patients had increased concentrations of hTERT mRNA in exosomes derived from their sera and a higher percentage of them had positive exosomal hTERT mRNA than healthy controls. This elevation was accentuated in stage IV disease patients and correlated with CEA levels but not with MSI status, which is in agreement with many previous studies demonstrating that hTERT expression and/or telomerase activity increase with tumor progression and are positively correlated with disease staging and poor differentiation, but not MSI status (6, 22, 23). Studies evaluating both tumor and plasma hTERT levels found a correlation between the two, and also higher circulating hTERT levels in stage 4 tumors (10, 24). In addition, hTERT’s prognostic role and association with worse overall survival have been found in CRC patients across all pathologic stages (6) and it was recently shown to be a useful marker for monitoring the therapy response in patients with rectal cancer undergoing neo-adjuvant chemoradiotherapy (11).

Still, most patients with non-metastatic CRC in our study did not secrete hTERT mRNA in their exosomes. Since the level of cellular telomerase activity in tumors and exosomal concentration of hTERT mRNA appear to be correlated (12), low levels of telomerase activity in early stage tumors, although not measured in this study, could have been be the cause of hTERT mRNA’s absence in exosomes. Alternatively, the content of exosomes is not merely a passive reflection of its constituents’ cellular concentrations. The involvement of the ‘endosomal sorting complex required for transport (ESCRT)’ complex in packaging nucleic acids in exosomes has been partially elucidated (14), and it may differ in various patients, depending on the cancer type and its expression.

Additionally, this study is the first to show that a significant subset of LS carriers without known active malignancies have elevated peripheral blood derived exosomal hTERT mRNA. In fact, both exosomal hTERT mRNA’s level and the proportion of patients with positive hTERT mRNA, were similar between LS- and non-metastatic CRC groups. There are several potential explanations for this interesting finding. First, although all LS patients had normal findings/benign polyps in their colonoscopy at the time of recruitment, and were all under routine follow-up for other at-risk organs, an occult malignancy could still occur, especially in organs which are not routinely surveilled, e.g., the urinary tract, small bowel or pancreas. Second, as recently reviewed by Ahadova et al., there are differences in the normal-appearing colonic mucosa between LS carriers and healthy individuals (25). Mismatch repair deficient crypt foci (MMR-DCF) can be found in the normal-looking colonic mucosa of LS carriers, which exhibit microsatellite instability (MSI) and carry mutations in microsatellite-bearing genes at very early tumorigenesis stages, even as an initiating event. These mutations result in the generation of frame-shift peptides (FSPs) that can elicit strong immune responses and elimination of FSP-expressing cells by T cells (26). Thus, continuous immunoediting, expressed by elevated mucosal T-cell infiltration and distinct immune profiles, constantly occur even in normal-appearing colonic mucosa (27). Moreover, systemic cellular immune responses to FSP have been found in the blood of healthy LS carriers (28). Exosomal telomerase mRNA can thus either reflect the perpetual generation and regression of MMR-deficient lesions, or originate from activated peripheral lymphocytes. Additionally, the status of the MMR system was previously shown to be important in defining telomere length, presenting by distinct patterns of peripheral blood telomere length in MMR-deficient and -proficient non-polyposis hereditary patients (19). It is possible that either high or low telomerase activity could be associated with shortened telomeres.

The significance and clinical consequences of high exosomal hTERT mRNA levels in LS-carriers are unknown. Although it is tempting to assume that these patients are at higher risk for malignancy, potentially needing stricter surveillance policy, we could not show this in our study since they were not followed up to see if they developed cancer. Moreover, exosomes can be a double-edged sword in cancer immunity. While they can transfer bioactive molecules between tumor cells to help cancer cells escape immune surveillance, those derived from immune cells can also inhibit tumor growth and proliferation (14). We did not find significant differences in the proportion of patients with positive hTERT mRNA among different MMR-gene variant carriers, despite the clinical and molecular differences between them, possibly due to the small sample size. A hint to hTERT’s potential clinical significance was shown in a large cohort of Dutch and Spanish LS carriers (20), in which polymorphism in the variant rs2075786 of the hTERT gene was associated with an increased risk of LS-related-cancers under 45 years of age, while not in the general population or in non-Lynch CRC families, making hTERT a potential cancer-risk modifier of LS.

In this study, two patients from the healthy control group had high exosomal telomerase. This could be attributed to an occult malignancy, either solid or hematological (e.g, lymphoma or chronic lymphocytic leukemia). Alternatively, other non-malignant conditions that are associated with activation of B- lymphocytes, e.g., metabolic syndrome (29, 30), systemic inflammatory disorders or mood disorders (31), could have been the cause of telomere attrition and the release of exosomal telomerase mRNA.

Our study has several limitations. First, the data from the medical files were collected retrospectively, which has an inherent bias. Second, the lack of recurrent measurements of exosomal- hTERT mRNA and long-term follow up of patients, precluded us from its evaluation as a prognostic marker or its association with treatment response. Third, cellular telomerase activity in tumors was not evaluated, thus we could not assess a tumor-exosome correlation of hTERT mRNA. Still, the findings of higher rate of positive exosomal hTERT mRNA in CRC patients which was stage-dependent, and in LS carriers without known malignancies, are innovative. While in CRC, it probably reflects the tumor burden and may have a prognostic effect, its significance and clinical applicability in LS is less clear, and more prospective studies with long-term follow-up and repeated telomerase testing should be performed to assess this.

## Data availability statement

The original contributions presented in the study are included in the article/supplementary material. Further inquiries can be directed to the corresponding author.

## Ethics statement

The study was approved by the Sheba Medical Center ethics committee and written informed consent was obtained from each patient included in the study. Approval was granted for Helsinki protocol 6423-19. The study protocol conforms to the ethical guidelines of the 1975 Declaration of Helsinki as reflected in *a priori* approval by the institution’s human research committee. The patients/participants provided their written informed consent to participate in this study.

## Author contributions

Conceptualization, IL and OU; methodology, IL, TB, and OU.; software, HM.; validation, HM and AA; formal analysis, HG.; investigation, OU; resources ZL, NH, EK, DF, and NS; data curation, OP, MY, EF, and EB; writing—IL, and OU; writing—review and editing, ZL, DF, HG, TB, and PR; visualization, PR; supervision, OU; project administration, IL; funding acquisition, IL and OU. All authors have read and agreed to the published version of the manuscript.

## Funding

This study was supported by research grants obtained from the Israeli Gastroenterology Society and from the Germanis Research Foundation affiliated with the CBRC at Tel-Aviv University, Israel.

## Conflict of interest

The authors declare that the research was conducted in the absence of any commercial or financial relationships that could be construed as a potential conflict of interest.

## Publisher’s note

All claims expressed in this article are solely those of the authors and do not necessarily represent those of their affiliated organizations, or those of the publisher, the editors and the reviewers. Any product that may be evaluated in this article, or claim that may be made by its manufacturer, is not guaranteed or endorsed by the publisher.
